# Transcription factor CASZ1 increases an oncogenic transcriptional process in tumorigenesis and progression of glioma cells

**DOI:** 10.1002/mco2.182

**Published:** 2022-10-20

**Authors:** Chaofu Mao, Chengying Huang, Zhicheng Hu, Shanqiang Qu

**Affiliations:** ^1^ Department of Neurosurgery Nanfang Hospital Southern Medical University Guangzhou Guangdong People's Republic of China; ^2^ Department of Obstetrics and Gynecology, Baiyun Branch Nanfang Hospital Southern Medical University Guangzhou Guangdong People's Republic of China; ^3^ Department of Burn Surgery First Affiliated Hospital Sun Yat‐sen University Guangzhou Guangdong People's Republic of China

**Keywords:** CASZ1, glioma, therapeutic targets

## Abstract

As a transcription factor, the role of CASZ1 in different entities is inconsistent. Glioma is one of the leading causes of cancer death worldwide. Its prognostic relevance and biological functions in glioma remain obscure. We focused on the role, mechanism, and prognostic value of CASZ1 in glioma cells. Herein, CASZ1 was identified as a novel potential oncogene in glioma tissues from GEO and TCGA datasets. CASZ1 was highly expressed in glioma tissues, predicting poor prognosis in glioma patients. Knockdown of CASZ1 inhibited proliferation and invasion in vitro, whereas upregulation of CASZ1 presented opposite results. Overexpression of CASZ1 increased transcriptional process of target gene p75NTR. CASZ1 was the potential transcriptional regulators for p75NTR. In addition, the p75NTR expression is essential for CASZ1 to exert its function as an oncogene. Our findings indicate that highly expressed CASZ1 in glioma cells acts as a pro‐oncogene factor in gliomas via regulating transcriptional process of target gene p75NTR, which was identified as an unfavorable prognostic marker in patients with gliomas. CASZ1 is expected to become a novel target for the treatment of gliomas.

## Graphical abstract

CASZ1 overexpressed is inversely correlated with the unfavorable prognosis of glioma patients, which was confirmed as a new predictive indicator. The CASZ1/p75NTR signaling axis is vital in the development and progression of glioma, being a novel potential target for glioma therapy. CASZ1 is hypo‐methylated in gliomas, and its status is negatively correlated with CASZ1 mRNA level.

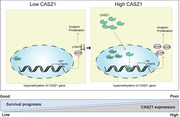

## 1 INTRODUCTION

Originating from progenitor glial cells or neural stem cells,^1^ glioma is the most frequent brain malignancy. Based on the latest epidemiological data, the global incidence of brain tumors is 5.48 per 100,000,^2^, ^3^ and the 1‐ and 5‐year survival rates are 39.5% and 5.4%, respectively. With the advance of diagnosis and therapy technology of gliomas, the current radiotherapy and chemotherapy were improved to certain extent in recent decades. However, the overall survival (OS) of patients remains unsatisfactory. Thus, it is very necessary to identify the molecular mechanism of gliomas and the novel molecular targets to improve the outcome of patients.

Using the microarray‐based analysis, we previously demonstrated that the transcription factor CASZ1 was significantly upregulated in gliomas. CASZ1 (also known as ZNF693) is a zinc finger gene family member and is localized to chromosome 1p36.22.^4^ CASZ1 is expressed in multiple tissues and it is involved in several biological processes, including cell differentiation and proliferation.^5^ Recently, Liu et al. confirmed that CASZ1 could suppress the growth and migration of neuroblastoma.^6^ Alternatively, an inverse trend was observed. However, CASZ1 was upregulated in epithelial ovarian cancer and promoted epithelial–mesenchymal transition (EMT).^7^ The controversial findings hamper our understanding of CASZ1 across diverse solid tumors, the biological function and clinical significance of CASZ1 in gliomas further remain obscure.

As a TNF superfamily, p75NTR (NGFR) is a transmembrane type Ⅰprotein with 427 amino acids, including a 28‐amino acid signal peptide. Previous literature has reported that p75NTR is expressed in many tumor tissues, such as gastric cancer, glioma, bladder cancer, and breast cancer.^8^ Griesmaier et al. reported that p75NTR protein can cooperate with Trk receptor to improve or reduce the responsiveness of neurotrophic factors and further regulate cell survival.^9^ Previous studies have found that neurotrophin stimulates the proliferation and migration of melanoma cells through its receptors Trk and p75NTR, and p75NTR can promote the survival of melanoma cells with brain metastases.^8^ Wang et al. observed that p75NTR could promote glioma cells invasion and progression in a ligand‐dependent manner.^10^ Nevertheless, the regulatory factors driving p75NTR expression in gliomas remain unclear.

In this study, CASZ1 was characterized. For the first time, we identified that the mRNA and protein levels of CASZ1 were significantly upregulated in glioma cells. CASZ1 overexpression had significant effects on tumorigenesis and progression of glioma cells. We investigated the hypothesis that transcriptional regulatory activation of the p75NTR gene could rescue these effects. In addition, *CASZ1* DNA demethylation was associated with gene upregulation, which promotes CASZ1 expression by methylation analysis. Our study indicated that CASZ1 might be a potential prognostic biomarker and a therapeutic target for gliomas.

## 2 RESULTS

### 2.1 Upregulated CASZ1 expression is associated with clinical features and patient prognosis in glioma

**FIGURE 1:**
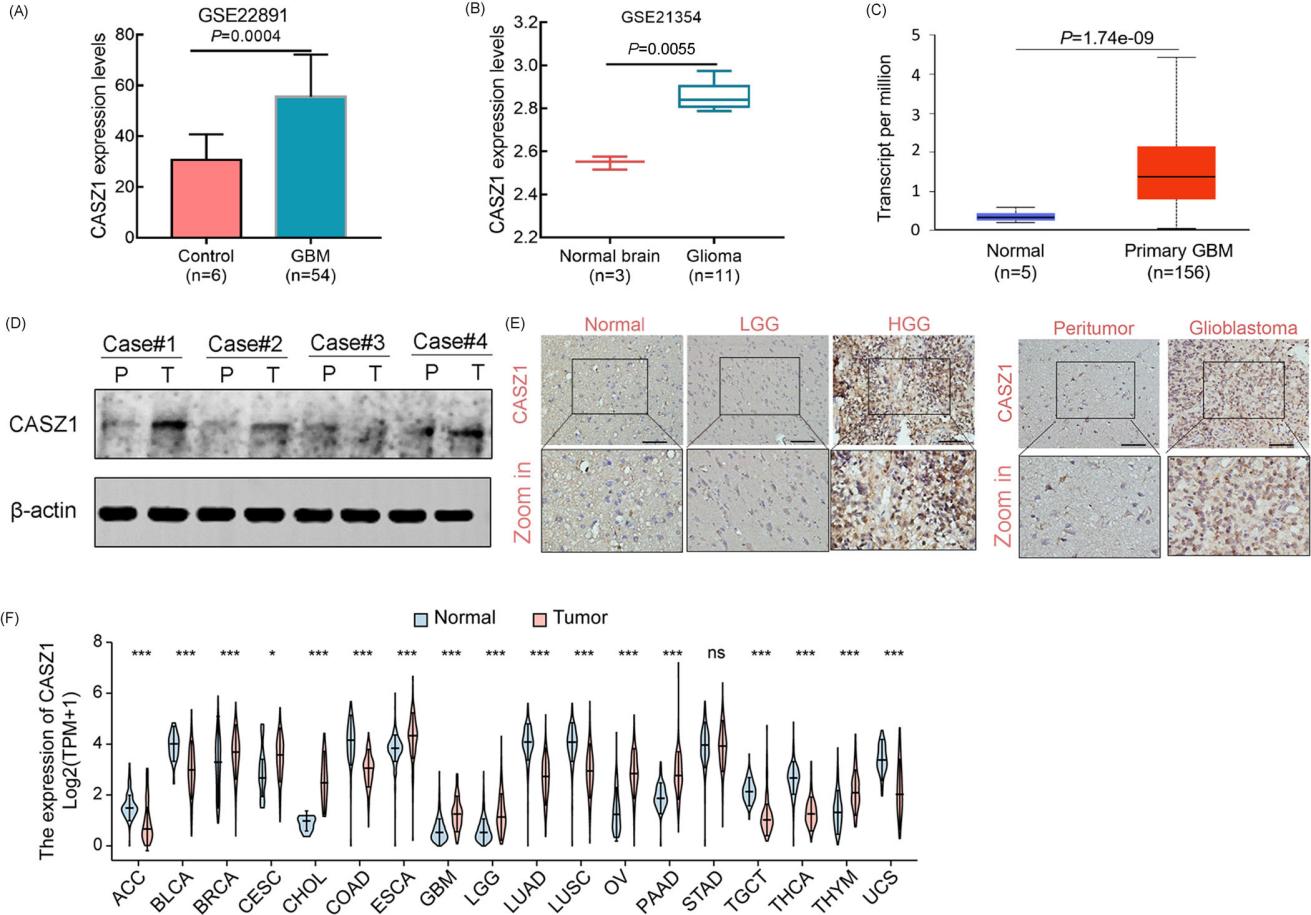
CASZ1 gene expression is upregulated in human gliomas. (A–C) The relative expression of CASZ1 was compared between normal brain tissues and glioma tissues in the GEO and TCGA datasets. (D) The relative expression of CASZ1 was determined in the pairs of glioma tissues (T) and peritumoral tissues (P) by Western blot. (E) The differential expression of CASZ1 in glioma and normal brain tissues was verified by immunohistochemistry. Scale bar = 100 μm. (F) The differential expression of CASZ1 between other solid tumors and normal control tissues was determined by TCGA datasets. HGG: high‐grade glioma; LGG: low‐grade glioma; GBM: glioblastoma

To explore the mRNA and protein expression patterns of *CASZ1* in glioma, GEO (GSE22891 and GSE21354) and TCGA datasets were accessed. We observed that *CASZ1* mRNA expression was overexpressed in gliomas compared to normal brain tissues (Figure 1A–C). These results were also confirmed in clinical glioma tissues by Western blot and immunohistochemistry (IHC) (Figure 1D,E). Additionally, we analyzed *CASZ1* expression patterns in other solid tumors from TCGA and observed that there was a degree of variability in the expression of *CASZ1* across different tumors (Figure 1F), which suggested that CASZ1 seemed to play distinct roles in the different tumor types.

**FIGURE 2:**
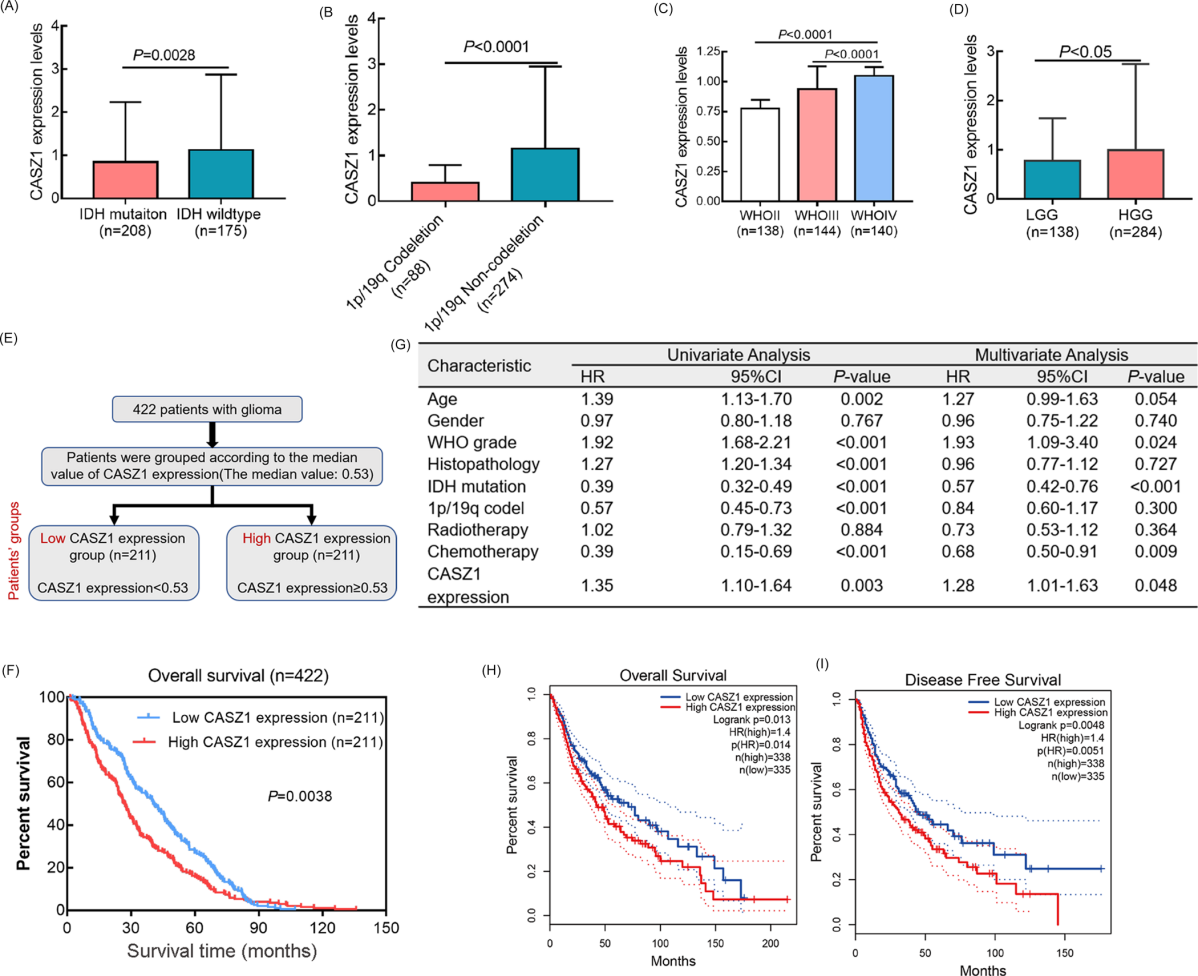
CASZ1 overexpression is significantly correlated with malignant progression and poor prognosis of glioma patients. (A–D) Dot distribution graphs of CASZ1 expression were shown in 422 glioma patients with different clinical features. (E) According to the median value of CASZ1 expression, each patient was assigned to the high and low CASZ1 expression groups. (F) Kaplan–Meier survival curve analysis showed that glioma patients with high expression of CASZ1 had shorter overall survival time. (G) Univariate and multivariate Cox regression analyses for prognosis in patients with gliomas. (H and I) The effects of CASZ1 on prognosis and progression‐free survival of glioma patients were verified by TCGA data.

Within the 422 glioma cases included in the CGGA cohort, *CASZ1* expression increased in glioma tissues with 1p/19q non‐codeletion, wild‐type IDH, and high grade groups (Figure 2A–D). Patients were then dichotomized into low‐*CASZ1* and high‐*CASZ1* groups (Figure 2E). The *CASZ1* expression was significantly associated with pathological type (*p* < 0.001), tumor grade (*p* < 0.001), IDH status (*p* = 0.037), and 1p/19q status (*p* < 0.001) (Table 1). Furthermore, patients with higher CASZ1 expression showed shorter OS than those with lower *CASZ1* expression (Figure 2F). Analysis through GEPIA yielded consistent results (Figure 2H,I). These data suggested that the overexpression of *CASZ1* might contribute to the malignant biological behavior. Univariate and multivariate Cox analyses confirmed *CASZ1* as a risk factor, potentially serving as a novel prognostic biomarker for gliomas (Figure 2G).

**TABLE 1:** Correlation between CASZ1 expression and clinicopathological characteristics of primary glioma patient (*n* = 422)

	CASZ1 expression		
Characteristics	Low expression	High expression	*p*‐Value
**Age**			
≥40	126	137	0.242
<40	85	73	
NA	0	1	
**Gender**			
Male	120	123	0.768
Female	91	88	
**WHO grade**			
WHO II	82	56	<0.001
WHO III	80	64	
WHO IV	49	91	
**Histopathology**			
O	17	6	<0.001
OA	46	31	
A	19	19	
AO	23	5	
AOA	43	39	
AA	14	20	
GBM	49	91	
**IDH**			
Mutation	109	99	0.037
Wildtype	73	102	
NA	29	10	
**1p/19q**			
Codel	64	24	<0.001
Non‐codel	110	164	
NA	37	23	
**Radiotherapy**			
Yes	150	163	0.224
No	49	38	
NA	12	10	
**Chemotherapy**			
Yes	141	140	0.825
No	54	61	
NA	16	10	

Abbreviations: A, astrocytoma; AA, anaplastic astrocytoma; AO, anaplastic oligodendroglioma; AOA, anaplastic oligoastrocytoma; GBM, glioblastoma; NA, not available; O, oligodendroglioma; OA, oligoastrocytoma.

### 2.2 CASZ1 overexpression promoted glioma cell proliferation and invasion

**FIGURE 3:**
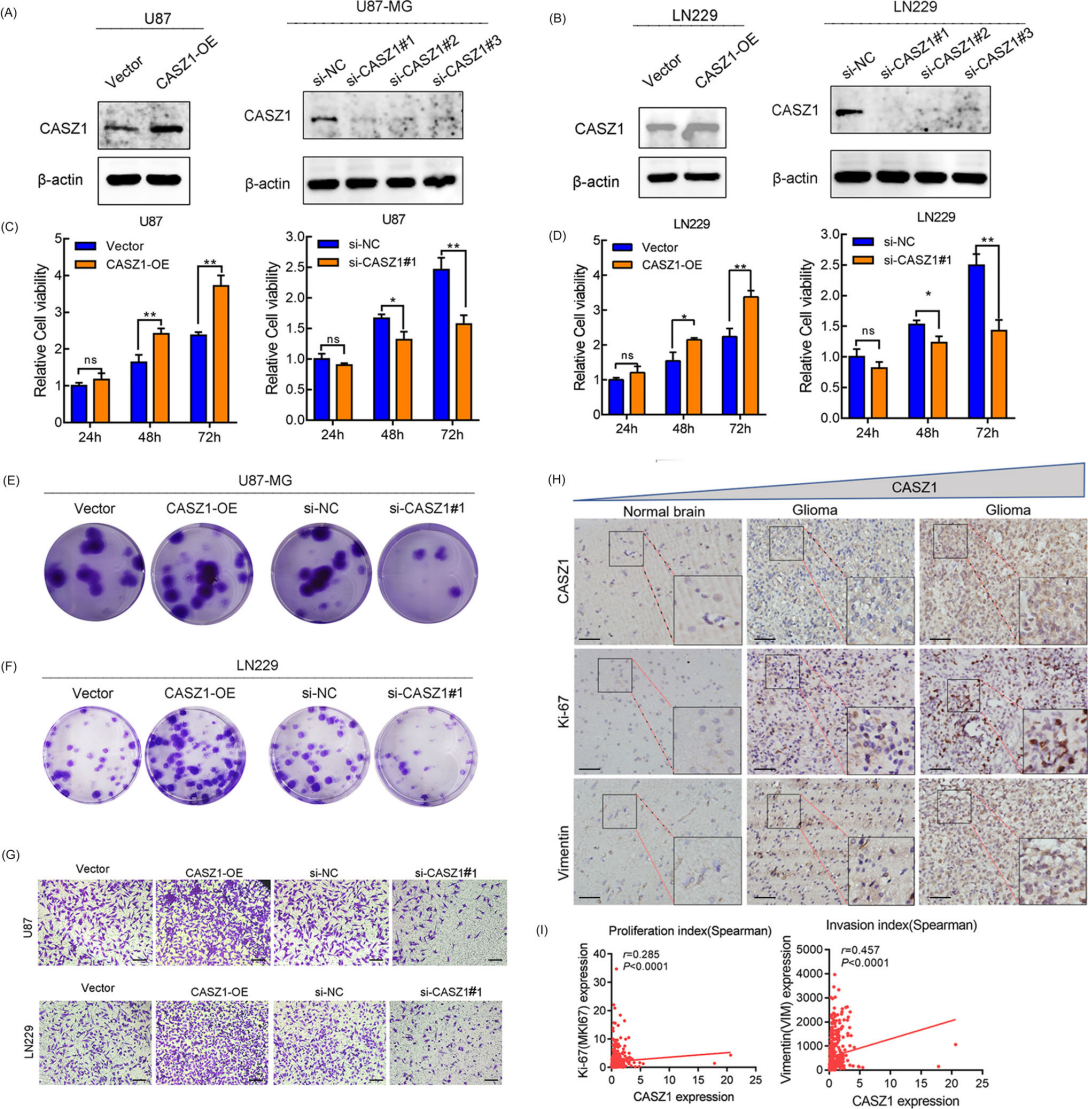
CASZ1 increases proliferation and invasion abilities of glioma cells. (A and B) The relative expressions of CASZ1 were detected in U87‐MG and LN229 cell lines after transfection with overexpressed CASZ1 plasmids and siRNA by Western blot. (C and D) CCK8 assay was used to assess glioma cell proliferation after transfection with overexpressed CASZ1 plasmids and siRNA. (E and F) Colony‐formation assay was performed to determine the proliferation ability of U87 and LN229 cells infected with si‐NC and si‐CASZ1#1. (G) Transwell assay of U87‐MG and LN229 cell lines’ invasive ability in different groups. Scale bar = 100 μm. (H) Association between CASZ1 and Ki‐67/vimentin expression in clinical glioma specimens. Scale bar = 100 μm. (I) Correlation of CASZ1 expression with proliferation and invasion indices in 422 glioma patients

Next, the biological role of CASZ1 overexpression in gliomas was investigated. Figure 3A,B shows CASZ1 protein levels after CASZ1 overexpression and knockdown in U87‐MG and LN229 cells (the first was the most efficient). The upregulation of CASZ1 could promote U87 and LN229 cells proliferation, whereas the knockdown could inhibit cell proliferation by CCK8 assays (Figure 3C,D). Additionally, plate clone assay results showed that CASZ1 expression positively regulated the proliferation of U87‐MG and LN229 cells (Figure 3E,F). Similarly, the upregulation of CASZ1 could promote cell invasion, whereas knockdown of which could suppress glioma cell invasion (Figure 3G). Using our own glioma specimens, we consistently observed significant correlations between CASZ1 protein expression and Ki‐67 and vimentin expression by IHC (Figure 3H). Within the CGGA cohort, *CASZ1* mRNA level was positively associated with tumor proliferation index Ki‐67 (*r* = 0.285, *p* < 0.0001) and invasion index vimentin levels (*r* = 0.457, *p* < 0.0001) (Figure 3I).

### 2.3 CASZ1 regulates the proliferation and invasion of glioma cells via promoting transcriptional process of oncogene p75NTR

**FIGURE 4:**
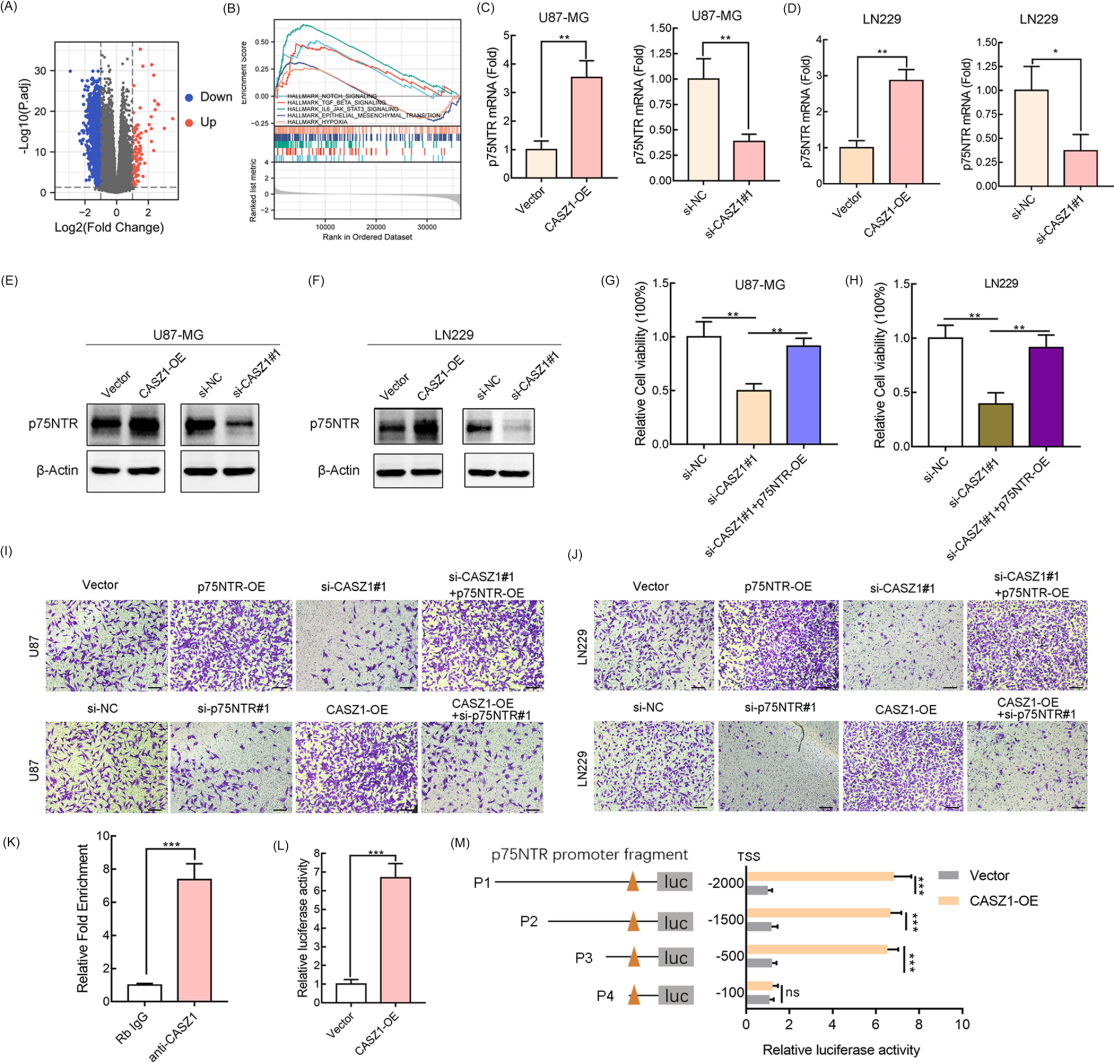
CASZ1 promotes the proliferation and invasion ability of glioma cells by upregulating the transcription process of p75NTR gene. (A) GSEA enrichment plots showed that enrichment of cancer‐related pathways was associated with upregulation of CASZ1. (B) Volcano map shows all the differential genes between high and low CASZ1 groups. (C–F) CASZ1 gene positively regulates the mRNA and protein levels of p75NTR gene was evident by qRT‐PCR and Western blot assays in U87‐MG and LN229. (G–J) CCK8 and transwell assays were used to validate that CASZ1 regulated the proliferation and invasion abilities of glioma cells through p75NTR. Scale bar = 100 μm. (K) ChIP‐qRT‐PCR assay performed using anti‐CASZ1 antibody. Rabbit IgG was used as a negative immunoprecipitation control. (L) Luciferase reporter assay of p75NTR transcriptional activity. (M) CASZ1 transcription factor increased p75NTR promoter activity by luciferase reporter assay. TSS: transcription start site

We further explored the molecular mechanism by which highly expressed CASZ1 promoted the malignant behaviors of glioma. First, the GSEA analysis revealed that *CASZ1* overexpression was related to cancer‐related signaling pathways, including the “Notch signaling,” “transforming growth factor‐beta (TGF‐β) signaling,” “IL6_JAK_STAT3 signaling,” and “EMT signaling” (Figure 4B). Besides, the GO and KEGG pathway enrichment analyses revealed that these genes were significantly involved in the immune‐related, inflammatory response and cancer pathways (Figure S1). We further analyzed the differentially expressed genes (DEGs) between the two groups (Figure 4A) and found that the *p75NTR* (*NGFR*) gene ranked top three among them by fold‐change filtering (Figure S2). Previous studies have proven p75NTR to be an oncogene in gliomas.^10^ Therefore, we speculated that CASZ1 could regulate downstream p75NTR expression. Quantitative reverse transcription‐polymerase chain reaction (qRT‐PCR) and Western blot revealed that CASZ1 positively regulated the mRNA and protein levels of p75NTR gene (Figure 4C–F). In addition, the cells proliferation and invasion abilities suppressed by si‐*CASZ1*#1 were rescued by the simultaneous overexpression of p75NTR (Figure 4G–J). Conversely, knocking down p75NTR could rescue the invasion abilities of glioma cells, which were promoted by overexpressed *CASZ1* gene (Figure 4I,J).

CASZ1 is a transcription factor, so does CASZ1 regulate the expression of p75NTR through transcription? We cloned the full‐length (FL) p75NTR promoter (2 kb) into a luciferase reporter plasmid. Chromatin immunoprecipitation assay (ChIP)‐qRT‐PCR assay confirmed that CASZ1 can directly associate with the p75NTR gene promoter (Figure 4K). Additionally, CASZ1 transcription factor was shown to drive a string transcription of p75NTR gene as confirmed by luciferase reporter assay (Figure 4L). The 2 kb‐FL promoter was fragmented into four segments (P1, from −2000 bp to transcription start site [TSS]; P2, from −1500 bp to TSS; P3, from −500 bp to TSS; P4, from −100 bp to TSS). As shown in Figure 4M, transcription factor binding site might be located within a region from −500 to −100 bp upstream the TSS of p75NTR. The above findings supported that CASZ1 directly regulated p75NTR transcription.

### 2.4 Association between CASZ1 expression and tumor microenvironment

**FIGURE 5:**
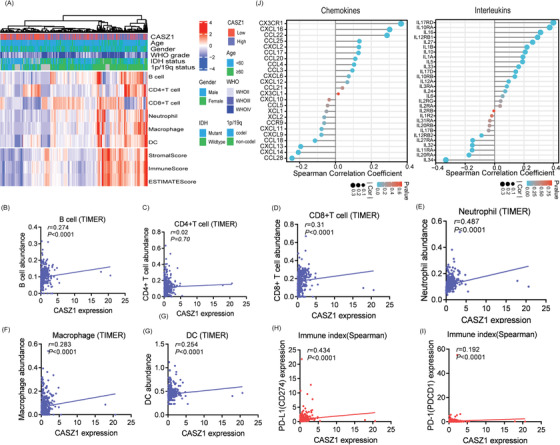
The difference in the relative abundance of immune infiltration in TME between low and high CASZ1 expression groups. (A) Patterns of immune cells in TME of each patient. (B–G) Correlation between CASZ1 expression and six immune cells infiltration by Spearman correlation analysis in glioma. (H–J) The CASZ1 expression was correlated with expressions of PD‐1/PD‐L1, chemokines, and interleukins.

Tumor‐infiltrating immune cells are strongly associated with tumorigenesis and progression.^11^ We noticed that overexpressed *CASZ1* was involved in cancer‐related signaling pathways and multiple immune‐inflammatory signaling pathways, including “B‐cell receptor signaling pathway,” “Toll‐like receptor signaling pathway,” “T‐cell receptor signaling pathway,” and “NF‐Kappa B signaling pathway” (Figure S1). Stromal score, immune score, and ESTIMATE score were calculated by the ESTIMATE algorithm^12^; and infiltration abundance was estimated using the TIMER algorithm.^12^ The immune landscape showed that diverse immune infiltration levels in the high‐*CASZ1* group were higher than those in the low group (Figure 5A). *CASZ1* expression was significantly correlated with the infiltrating abundances of B cell (*r* = 0.274, *p* < 0.0001), CD8^+^ T cell (*r* = 0.31, *p* < 0.0001), neutrophil (*r* = 0.487, *p* < 0.0001), macrophage (*r* = 0.283, *p* < 0.0001), and dendritic cell (*r* = 0.254, *p* < 0.0001) (Figure 5B–G). Moreover, the expression of *CASZ1* was also correlated with that of PD‐1 (*r* = 0.192, *p* < 0.0001) and PD‐L1 (*r* = 0.434, *p* < 0.0001) (Figure 5H,I).

Previously, we found that *CASZ1* overexpression was involved in TGF‐β signaling. TGF‐β inhibits the production of pro‐inflammatory cytokines. Thus, we also evaluated the correlation between expression of *CASZ1* and levels of chemokines and interleukins (Figure 5J). In gliomas with high *CASZ1* expression, we found a restricted infiltration level but upregulated chemokines, including CXCL16, CCL22, CCL25, and CXCL2, which could attract DCs and T cells.

### 2.5 Hypomethylation of CASZ1 is associated with CASZ1 gene expression and patient prognosis

**FIGURE 6:**
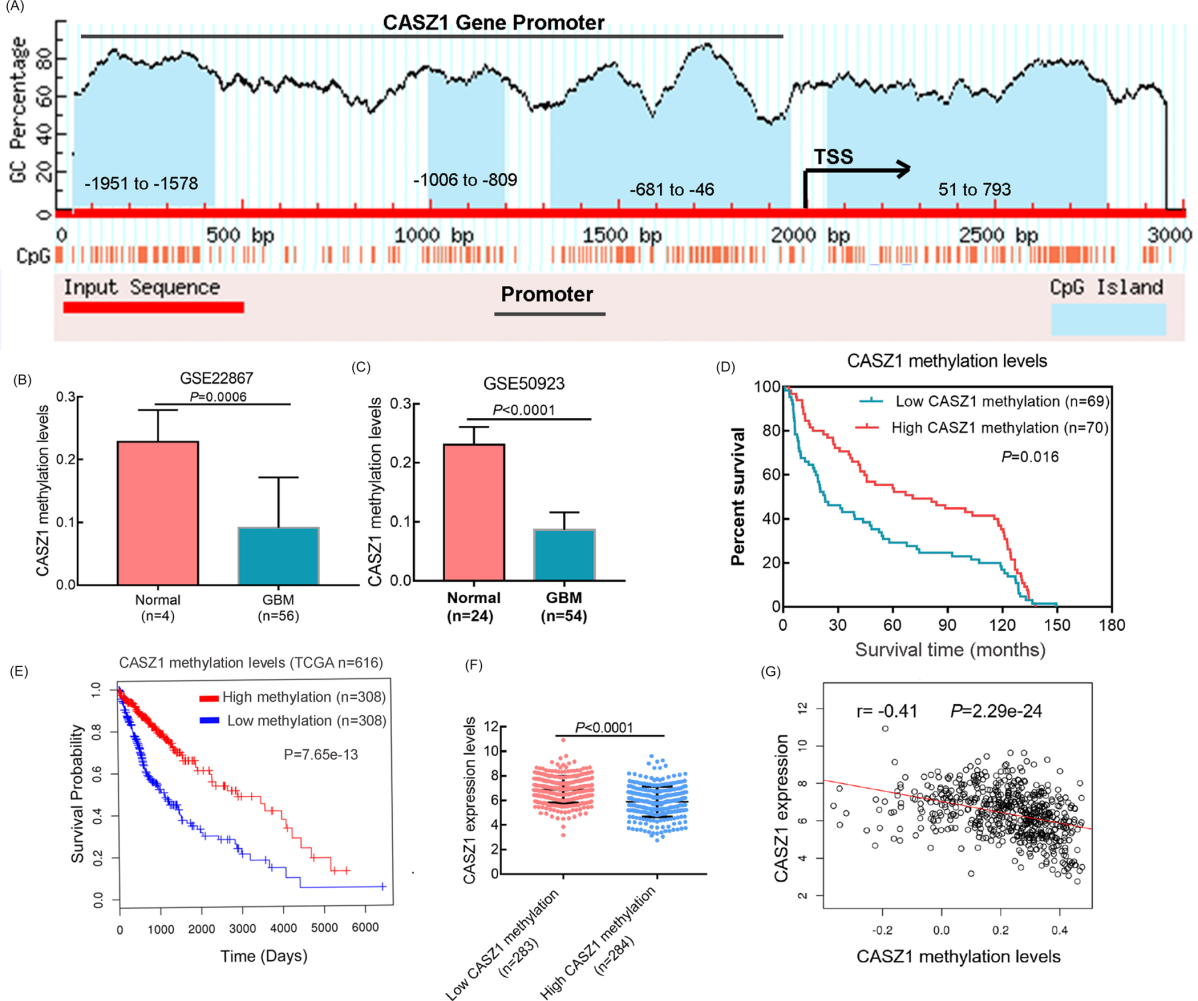
DNA methylation correlates inversely with CASZ1 expression and affects the outcome of glioma patients. (A) Distribution of CASZ1 methylation island in promoter region. (B and C) CASZ1 methylation data were obtained from GEO datasets. (D) The effect of CASZ1 gene methylation on prognosis was analyzed by Kaplan–Meier survival curve in 139 glioma patients. (E) The effect of CASZ1 gene methylation level on prognosis of patients was verified by TCGA data. (F and G) Differentially methylation genes negatively correlated with CASZ1 expression level.

Previous studies have confirmed that the methylation degree of gene promoter is closely related to its expression. Figure 6A shows the promoter region of the *CASZ1* gene, and the blue part is the CpG island. First, we analyzed two external GEO datasets, GSE22867 and GSE50923, and found that the methylation level of the *CASZ1* gene in glioma was remarkably decreased compared with normal brain tissue (Figure 6B,C). To determine the correlation between *CASZ1* methylation and clinicopathological features, we screened 139 cases of non‐recurrent glioma from the CGGA glioma database with methylation data. *CASZ1* methylation was significantly associated with the WHO grade and tumor histopathology (Table 2). Through the TCGA dataset (TCGA‐GBMLGG), we also verified that *CASZ1* mRNA expression was inversely correlated with its methylation levels (Figure 6F,G). The Kaplan–Meier analysis was performed to determine whether *CASZ1* methylation was associated with the prognosis of patients with gliomas. Low *CASZ1* methylation levels were associated with poor patient survival, using the log‐rank test (Figure 6D, *p* = 0.018), which was also verified in external cohort (*p* < 0.0001, Figure 6E).

**TABLE 2:** Correlation between CASZ1 methylation levels and clinicopathological characteristics of primary glioma patient (*n* = 139)

	CASZ1 methylation levels		
Characteristics	Low levels	High levels	*p*‐Value
**Age**			
≥40	33	33	0.871
<40	35	37	
NA	1	0	
**Gender**			
Male	46	36	0.068
Female	23	34	
**WHO grade**			
WHO II	15	45	<0.001
WHO III	25	15	
WHO IV	29	10	
**Histopathology**			
O	3	10	<0.001
A	12	35	
AO	7	5	
AOA	9	5	
AA	9	5	
GBM	29	10	

Abbreviations: A, astrocytoma; AA, anaplastic astrocytoma; AO, anaplastic oligodendroglioma; AOA, anaplastic oligoastrocytoma; GBM, glioblastoma; NA, not available; O, oligodendroglioma.

## 3 DISCUSSION

Despite the clinical administration of various agents, the high mortality rate of glioma remains a severe threat to patients’ lives and health worldwide.^13^ Thus, it is of great significance to further explore the molecular mechanism and potential novel markers of gliomas. We have determined the effect of the CASZ1/p75NTR signaling axis on the malignant biological behavior of glioma cells. We show that CASZ1 expression is significantly elevated in gliomas, and it contributes to the malignant properties of glioma cells through promoting the transcription process of p75NTR gene. The overexpression of CASZ1 protein may be caused by the low methylation abundance of the *CASZ1* gene, which was also considered a potential marker for glioma patient prognosis.

As a transcription factor, CASZ1 is widely expressed in eukaryotes and plays a crucial role in multiple developmental procedures, such as neurogenesis and cardiac development.^4^ It has been reported in the literature previously that CASZ1 expression in different solid tumors has different expression trends. Liu et al. observed that CASZ1 was significantly downregulated in neuroblastoma, and was identified as a tumor suppressor to suppress cell motility and proliferation via restoring pRB activity.^6^ Furthermore, Wang et al. reported that CASZ1 was less expressed in hepatocellular cancer tissues, and it suppressed the abnormal proliferation of tumor cells by affecting MAPK/ERK signaling, MMP2 and MMP9 expression in vitro,^14^ which was not consistent with our findings. The downregulated CASZ1 exert tumor‐suppressive functions via regulating oncogenes in neuroblastoma and hepatocellular cancer. However, we found that the overexpression of CASZ1 promoted glioma cells initiation and progression via activating the transcription of p75NTR gene. The p75NTR is a multiple function receptor and affects the proliferation, apoptosis, and survival of glioma cells, which was proposed to be a prospective anticancer target.^15^ These data suggest that CASZ1 might play a dual biological function in different tumors. Of note, Wu et al. also observed that CASZ1 was upregulated in ovarian malignancy and promoted tumor invasion and migration,^7^ though the underlying mechanism remains unknown. The role of CASZ1 in malignancies remains complex, which may be associated with tumor and tissue specificity.^7^, ^16^ Nevertheless, the expression pattern and functional mechanism of CASZ1 are seldom explored in gliomas. We demonstrate that CASZ1 expression is elevated in glioma tissues and cells. And upregulated CASZ1 promoted the malignant properties of gliomas. In contrast, the knockdown of CASZ1 led to opposing effects, consistent with the findings in ovarian cancer. Survival analyses suggested that CASZ1 independently predicted patient prognosis, as high CASZ1 expression always indicated a shorter OS. Although we had identified that CASZ1 was regulated and its potential mechanism, the factors driving CASZ1 expression remain poorly characterized. Further studies revealed that DNA methylation alterations might influence its expression.^17^

Typically, DNA methylation occurs at CpG sites. DNA that has been methylated is recognized by specific proteins that bind to it.^18^ After binding with a DNA‐binding protein, the DNA strand is tightly arranged, and the transcription factor binding and subsequent RNA synthesis are prevented.^19^ Therefore, DNA methylation inhibits the transcription process of genes. Wang et al. reported that *CASZ1* was significantly hypermethylated, which could lead to gene silencing and promote the occurrence of esophageal squamous cell carcinoma based on extracted cell‐free plasma DNA.^20^ Our study found low methylation level of the *CASZ1* gene in glioma and an evident negative correlation between methylation level and expression. This as well explains the upregulated CASZ1 mRNA and protein levels in gliomas. Epigenetic modification plays an important role in the development of gliomas, where DNA methylation is a common method of regulating transcription and correlates with apoptosis and cell cycle.^21^ Four methylation biomarkers based on sputum from patients with lung cancer contribute to early cancer screening.^22^ Similarly, this study demonstrated that CASZ1 expression might be regulated by DNA methylation, and higher methylation levels were related to a better outcome in glioma patients. This provides new insight into the prognosis prediction and treatment for patients with glioma. A few shortcomings also exist in this study. First, we have not fully elucidated the molecular mechanism of CASZ1 methylation. Second, this study was not verified by xenograft tumor model.

## 4 CONCLUSIONS

In summary, this study reports that CASZ1 is overexpressed in gliomas, and is inversely correlated with the unfavorable prognosis of glioma patients, which was confirmed as a new predictive indicator. Transcription factor CASZ1 could directly regulate the expression of target genes by binding to the p75NTR gene promoter. The CASZ1/p75NTR signaling axis is vital in the development and progression of glioma, being a novel potential target for glioma therapy. Besides, CASZ1 expression was related to levels of immune infiltration and chemokines in gliomas. CASZ1 is hypomethylated in gliomas, and its status is negatively correlated with CASZ1 mRNA level.

## 5 MATERIALS AND METHODS

### 5.1 Cell culture and transfection

We purchased glioma cell lines from the American Type Culture Collection (Manassas, VA, USA). They were cultured in DMEM (high glucose, BI, Israel) supplemented with 10% fetal bovine serum (BI, Israel) and 1% streptomycin‐penicillin (BI, Israel). Glioma specimens were obtained from the Nanfang Glioma Center. The transient expression of CASZ1 or p75NTR was achieved by transfecting cells with CASZ1 or p75NTR‐expressing plasmids. The transfection procedures were performed according to the manufacturer's protocol. Detailed steps are described in Supporting Information (Materials and Methods section).

### 5.2 Data source

The clinicopathological information and RNA expression data were downloaded from the Chinese Glioma Genome Atlas (CGGA).^23^, ^24^ Four hundred twenty‐two patients with glioma were included after excluding those with recurrent glioma. Their detailed demographics are summarized in Table S1. According to previous reports,^25^, ^26^ stromal and immune abundance in gliomas were estimated via the ESTIMATE algorithm. And we used the tumor immune estimation resource (TIMER) algorithm to assess immune infiltration levels. Next, we analyzed the effect of CASZ1 on patient survival outcome using the GEPIA^27^ and LinkedOmics databases.^28^

### 5.3 Immunohistochemistry

Immunohistochemistry was previously described elsewhere.^29^ In brief, we sliced paraffin‐embedded tissue blocks into 3 μm sections, deparaffinized and rehydrated them. Pressure cooking with EDTA buffer (pH 8.0) for 15 min was performed for antigen retrieval. We blocked endogenous peroxidase with 0.3% H_2_O_2_. Subsequent to the blocking with 5% bovine serum albumin for 45 min, sections were incubated with CASZ1 antibody (1:50, Cat No. SAB43214), Ki‐67 (1:200, Cat No. SAB5500134), vimentin (1:300, Cat No.bs‐0756R), and secondary antibody. We covered the resulting sections with diaminobenzidine to visualize staining and counterstained them with hematoxylin.

### 5.4 Cell proliferation and invasion assays

We used Cell Counting Kit‐8 (CCK8, Ribobio, China) and plate clone assay for cell proliferation tests. The detailed steps for CCK8 assay were described in previous study.^30^ Briefly, after digestion, 1 × 10^3^ cells were plated into the 96‐well plate for five replicate wells. The 96‐well plates were placed in the incubator at 37°C for the indicated time. Ten microliters of CKK8 solution was added to the 96‐well plate according to the CCK8 kit instructions. The optical density (OD) was measured at a wavelength of 450 nm. We precoated transwell chambers (Costar, USA) with Matrigel (R&D Systems, USA) for subsequent cell invasion assessment. A total of 1 × 10^5^ cells were plated in the upper chamber (8 mm, Coring Inc.). DMEM supplemented with 10% FBS was plated in the lower chambers. Following incubation for 24 h, migratory cells in the lower membrane were fixed with 4% paraformaldehyde for 30 min. After washing three times with PBS, the cells were stained with 0.4% crystal violet solution. The cells were once washed again with PBS and air‐dried.

### 5.5 Western blot

Western blot was carried out as previously described.^29^ Total proteins were extracted with precooled RIPA buffer supplemented with 1% protease inhibitors cocktail and 1% phosphoric acid protease inhibitor, and then quantified by the BCA assay (Beyotime Inc., China). After protein samples, we used 10% SDS‐PAGE gel electrophoresis to subject the extracted proteins. Subsequently, samples were transferred to the PVDF membranes (Invitrogen, Carlsbad, CA, USA). After washing with 1XTBST, the PVDF membranes were blocked with 5% BSA and then incubated with primary antibodies overnight at 4°C. The primary antibodies used in this study included CASZ1 (1:50, Cat No. SAB43214), p75NTR (1:1000, Cat No. 8238), and β‐actin (1:3000, Cat No. ab8227) antibodies. The following day, membranes were washed with 1XTBST three times, and secondary antibody was incubated for 1 h. We utilized enhanced chemiluminescence (ECL) kit to visualize the target bands, which were quantified by the ECL system (Pierce, USA).

### 5.6 Luciferase reporter assay

Dual‐Luciferase Reporter Assay System was applied for luciferase reporter assay. First, we cloned the 2 kb sequences of p75NTR promoter from human genomic DNA into plasmid pGL4.10 (Promega, #E6651) by PCR assay. Truncated promoter fragments were generated by FL promoter via PCR assay. Second, in luciferase reporter assay, 1 × 10^5^ HKE293T cells were plated onto 24‐well plates before transfection. The next day, cells were co‐transfected with a firefly luciferase reporter vector based on control pcDNA3.1 empty vector (EV, 225 ng) or wild‐type CASZ1 plasmid (225 ng), and a henifella luciferase control vector pGL4.73 (50 ng, Promega). After transfection, cell lysates were prepared and the values of firefly/Henilla luciferase were quantified using the Firely and Renilla dual luciferase assay (Biotium, #30005‐2) on a Glomax 96‐plate reader (Promega).

### 5.7 Chromatin immunoprecipitation assay

ChIP and qRT‐PCR assay was conducted as previously descried.^31^ In brief, immunoprecipitation of CASZ1 antibody was obtained by incubating the supernatant with CASZ1 antibody or normal rabbit IgG overnight at 4°C after formaldehyde cross‐linking and sonication of HKE293T cells. The mixture was then incubated with protein G‐Sepharose beads (GE Healthcare) at 4°C for 2 h and washed with radio‐immunoprecipitation assay (RIPA) buffer, lithium buffer, and Tris‐EDTA (TE) buffer. The cross‐linked DNA–protein complex was reversed through incubation at 65°C overnight. Immunoprecipitated DNA was ethanol precipitated. The fold enrichment relative to the IgG control was measured by qPCR.

### 5.8 Statistical analysis

All data were analyzed by SPSS version 23.0, Graph Pad Prism version 7.0, and R language version 3.6.1. The data were presented as mean ± standard deviation (SD). We used the student's *t*‐test to examine the statistical differences between two groups, and one‐way analysis of variance for three or more groups. Survival curves were assessed using Kaplan–Meier method and log‐rank test. The chi‐square (or Fisher's exact) tests were used to seek differences within categorical variables. Two‐tailed *p* < 0.05 was statistically significant.

## AUTHOR CONTRIBUTIONS

Shanqiang Qu, Chengying Huang, and Zhicheng Hu conceived and supervised this study. Chaofu Mao and Chengying Huang wrote the manuscript. Shanqiang Qu and Chaofu Mao conducted the data analysis. Zhicheng Hu and Chaofu Mao conducted the experiments. All authors have read and approved the final manuscript.

## CONFLICT OF INTEREST

The authors declare that there is no conflict of interest.

## ETHICS STATEMENT

This study was approved by the Ethics Committee of Nanfang Hospital. Patient tumor samples were obtained by informed consent.

## Supporting information

Supporting Information

## Data Availability

All data are available from the corresponding authors upon request.
