# Supplementary material for: Transcription factor CASZ1 increases an oncogenic transcriptional process in tumorigenesis and progression of glioma cells
Source: MedComm (2020). 2022 Oct 20;3(4):e182. doi: 10.1002/mco2.182 (PMC9583698; doi:10.1002/mco2.182)
Supplement: Supplementary file 1 — Supporting Information [file MCO2-3-e182-s001.docx]

***Supplementary Data***

**Transcription factor CASZ1 increases an oncogenic** **transcriptional process in tumorigenesis and progression of glioma cells**

Chaofu Mao ^1, #^, Chengying Huang^2, #^, Zhicheng Hu^3, #^ and Shanqiang Qu^1,^ *

^1^ Department of Neurosurgery, Nanfang Hospital, Southern Medical University, Guangzhou 510515, Guangdong, People’s Republic of China

^2^ Department of Obstetrics and Gynecology, Baiyun Branch, Nanfang Hospital, Southern Medical University, Guangzhou 123123, People’s Republic of China

^3^ Department of Burn surgery, first Affiliated hospital, Sun Yat-sen University, Guangzhou 510080, Guangdong, People’s Republic of China

- **Supplementary Table**

Table S1. Demographic and clinicopathological features of glioma patients (n=422).

| **Features** | **Category** | **Number of cases** | **Percentage (%)** |
| --- | --- | --- | --- |
| **Age (years)** | <40 | 158 | 37.5 |
|  | ≥40 | 263 | 62.3 |
|  | NA | 1 | 0.2 |
| **Sex** | Male | 243 | 57.6 |
|  | Female | 179 | 42.4 |
| **WHO grade** | WHO-grade II | 138 | 32.7 |
|  | WHO-grade III | 144 | 34.1 |
|  | WHO-grade IV | 140 | 33.2 |
| **Histopathology** | O | 23 | 5.5 |
|  | OA | 77 | 18.2 |
|  | A | 38 | 9.0 |
|  | AO | 28 | 6.6 |
|  | AOA | 82 | 19.4 |
|  | AA | 34 | 8.1 |
|  | GBM | 140 | 33.2 |
| **IDH** | Mutation | 208 | 49.3 |
|  | Wild-type | 175 | 41.5 |
|  | NA | 39 | 9.2 |
| **1p/19q** | Codel | 88 | 20.9 |
|  | Non-codel | 274 | 64.9 |
|  | NA | 60 | 14.2 |
| **Radiotherapy** | Yes | 313 | 74.2 |
|  | No | 87 | 20.6 |
|  | NA | 22 | 5.2 |
| **Chemotherapy** | Yes | 281 | 66.6 |
|  | No | 115 | 27.2 |
|  | NA | 26 | 6.2 |

O: oligodendroglioma; OA: oligoastrocytoma; A: astrocytoma; AO: anaplastic oligodendroglioma; AOA: anaplastic oligoastrocytoma; AA: anaplastic astrocytoma; GBM: glioblastoma.

- **Supplementary Figures**

**Figure S1**


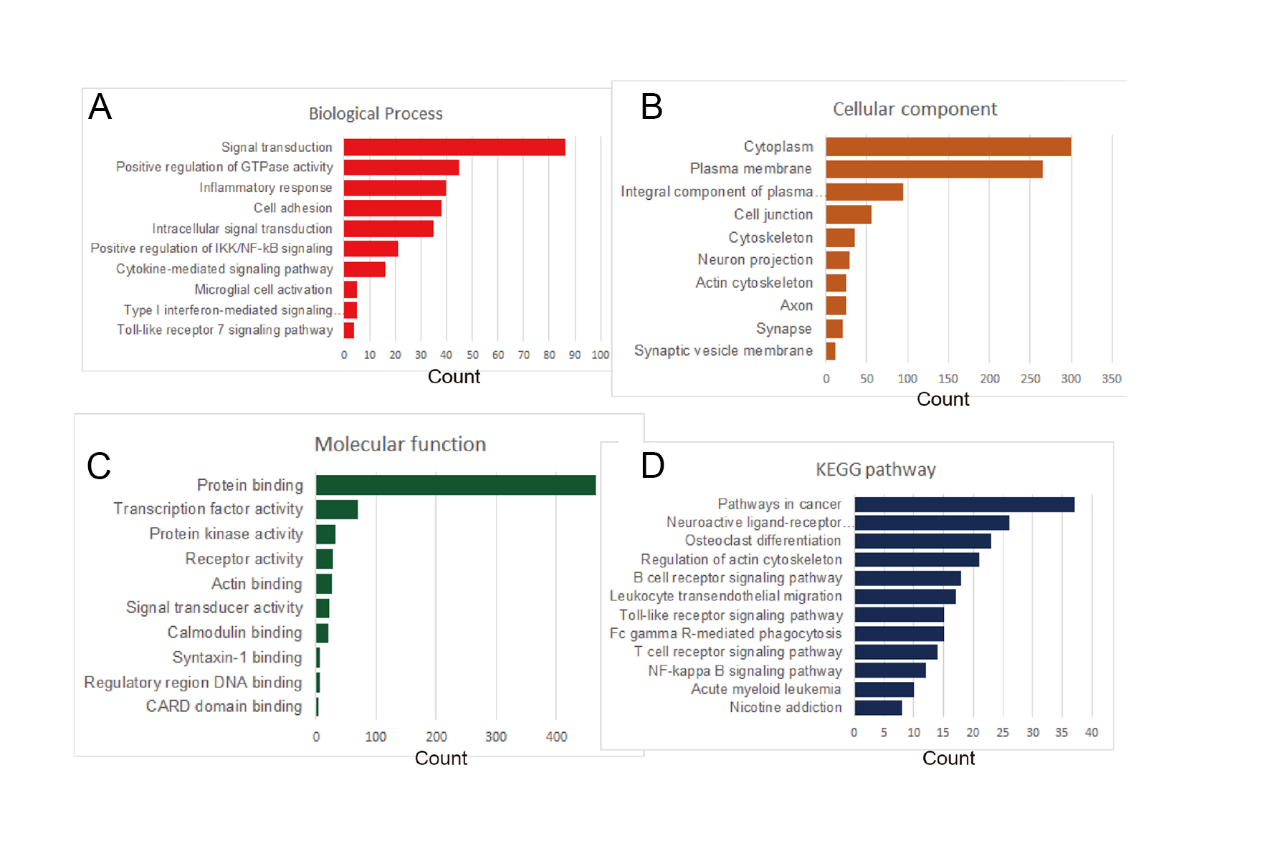


Figure S1. Enrichment of gene ontology analysis and KEGG pathway analysis (GO top 10 Go-terms and KEGG top 12 pathways). A: GO biology process; B: GO cellular component; C: GO molecular function; and D: KEGG pathway annotation.

**Figure S2**


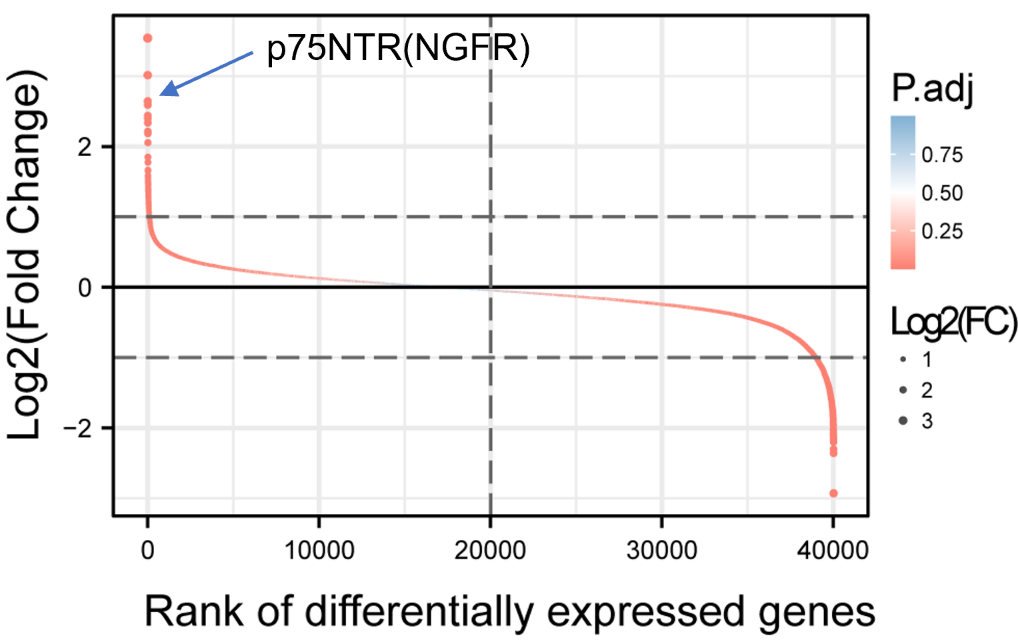


Figure S2. In glioma patients, the genes are co-expressed with CASZ1 gene.

- **Supplementary Materials and Methods**

1. **Cell Transfection**

After plating cells in 6-well plates, cells were transfected 24 hours later. Plasmid DNA (4.0ug) was diluted in 250ul of Opti-MEM I Reduced Serum per well. A diluted solution of Lipofectamine 2000 reagent (10ul) was prepared using Opti-MEM I (250ul) per well. After mixing the DNA and Lipofectamine mixtures, they were left to stand for 20 minutes at room temperature. The transfection reagent/DNA mixture was added dropwise to each well of the 6-well plate. The medium was replaced 8-12 hours after transfection. Western blot was used to verify the transfection efficiency after 48 hours. Cells were transfected with siRNA using lipofectamine 2000 according to the manufacturer's instructions. Shanghai Genechem Biotechnology Co., Ltd. provided the negative control siRNA and CASZ1/p75NTR siRNA. We used the p75NTR siRNA with the highest knockdown efficiency reported in previous literature. p75NTR siRNA #1, GGACAGAGUCUGGGUGUAUUUAUUU (sense) and AAAUAAAUACACCCAGACUCUGUCC (antisense).

1. **RNA Extraction and Quantification**

As previously described, total RNA was extracted, quantified, and cDNA was prepared^1^. Human p75NTR forward primer CCTGCCTGGACAGTGTG and reverse primer TCAGGTGCCACGGCTCAC. The comparative Ct values method was used to calculate the relative quantity.

- **Supplementary Western Blot Raw data**


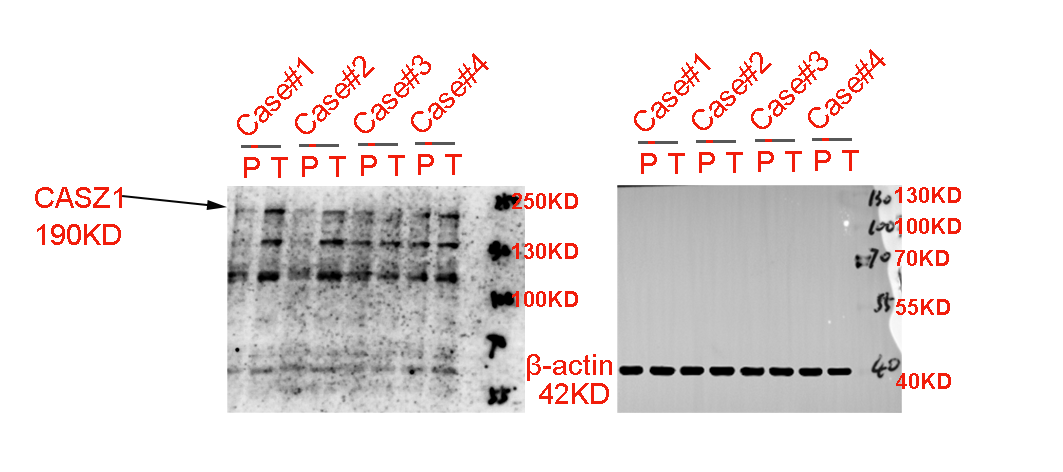


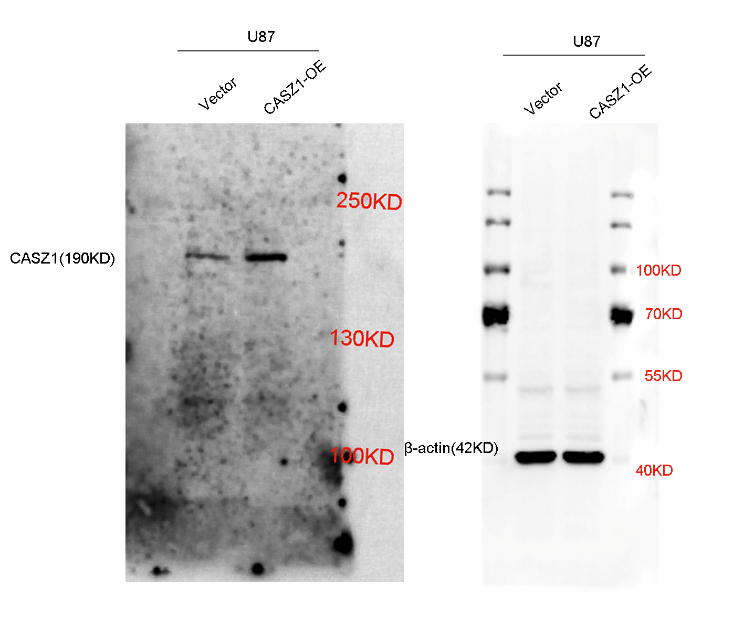


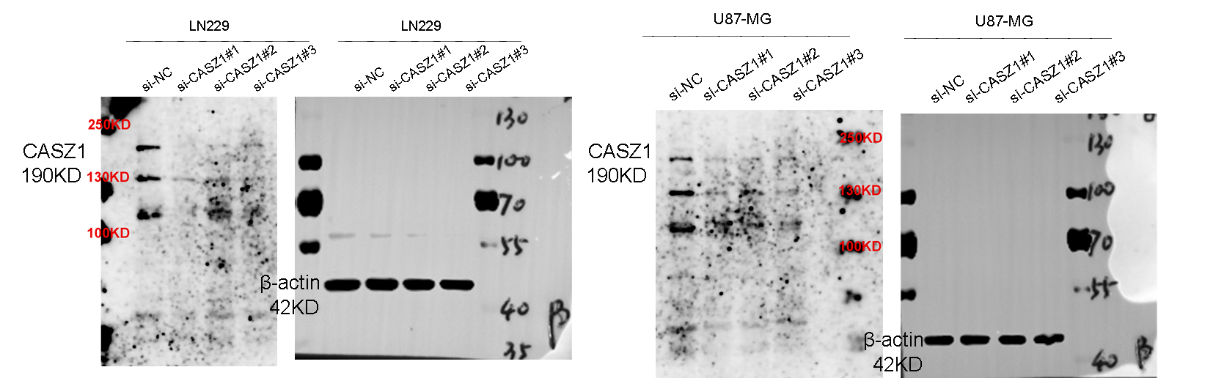


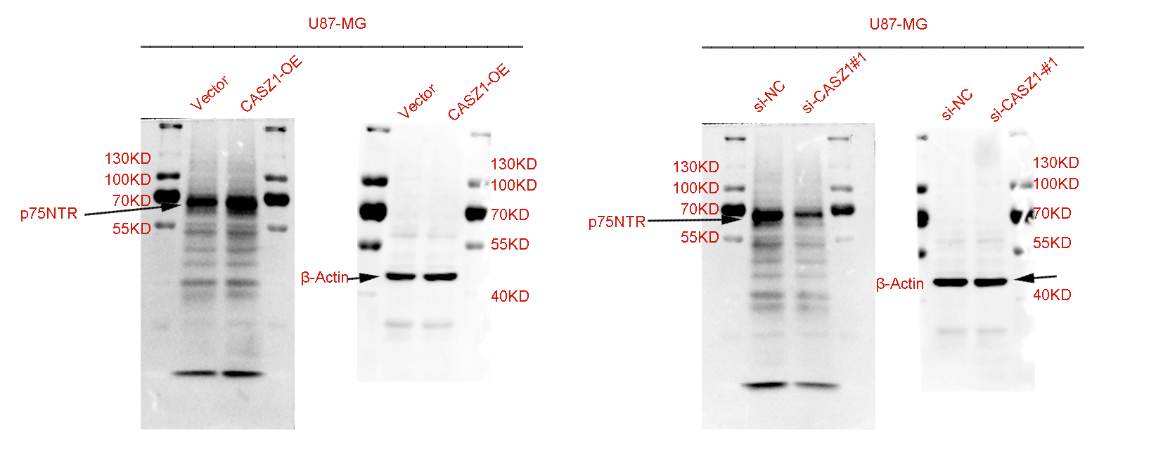


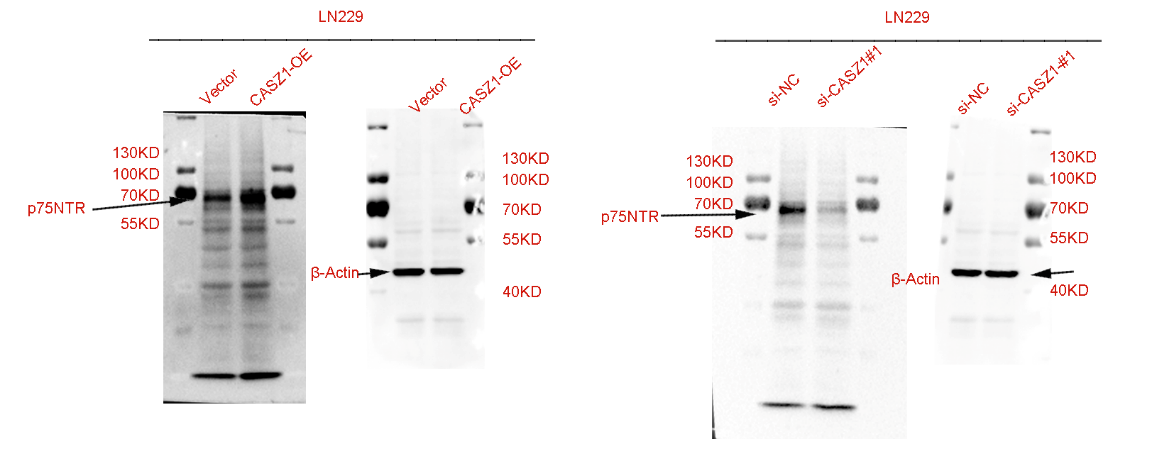


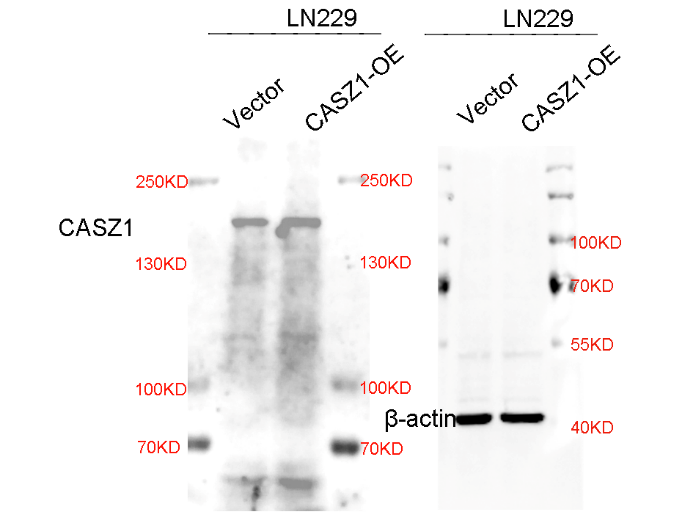


1. Qadir, A.S., Ceppi, P., Brockway, S., et al. CD95/Fas Increases Stemness in Cancer Cells by Inducing a STAT1-Dependent Type I Interferon Response. *Cell reports*. 2017;18(10):2373-2386.
